# The Preservation of Bone Cell Viability in a Human Femoral Head through a Perfusion Bioreactor

**DOI:** 10.3390/ma11071070

**Published:** 2018-06-25

**Authors:** Aparna Swarup, Hilary Weidner, Randall Duncan, Anja Nohe

**Affiliations:** 1 Department of Biological Sciences, University of Delaware, 105 The Green, Newark, DE 19716, USA; aswarup@udel.edu (A.S.); anjanohe@udel.edu (R.D.); rlduncan@udel.edu (A.N.); 2 Department of Biomedical Engineering, University of Delaware, 105 The Green, Newark, DE 19716, USA

**Keywords:** bioreactor, perfusion, femoral head

## Abstract

Current methods for drug development and discovery involve pre-clinical analyses that are extremely expensive and time consuming. Animal models are not the best precedent to use, when comparing to human models as they are not synonymous with the human response, thus, alternative methods for drug development are needed. One of which could be the use of an ex vivo human organ where drugs could be tested and the effects of those drugs could be observed. Finding a viable human organ to use in these preliminary ex vivo studies is difficult due to the availability, cost, and viability. Bone tissue and marrow contain a plethora of both bone and stem cells, however, these cells need constant perfusion to be viable over a longer time range. Here we maintain bone cell sustainability in an ex vivo model, through the use of human femoral heads in a novel bioreactor. This bioreactor was designed to directly perfuse cell culture media (DMEM) through the vasculature of a femoral head, providing ideal nutrients and conditions required for maintaining organ viability. We show, for the first time, that cells within a femoral head can stay alive up to 12 h. Further development could be used to determine the effects of drugs on a human organ system and could aid in the understanding of the progression of bone diseases and pathologies.

## 1. Introduction

Pre-clinical studies for drug development are time consuming, expensive, and the number of animals’ lives lost in the process is astronomical. In general, animal models are not optimal models, because they are not synonymous with the anatomy and physiology of humans. There is a need for another model for drug development. The human skeletal system could be the optimal model because bones contain a plethora of stem and bone cells. The skeleton also contains bone marrow, which is sensitive to toxicity. Bone tissue is very structurally sound and rigid, therefore, there is no need for additional external support that would be required for other organs. Testing drugs on a human femoral head ex vivo, would be ideal because the direct effects could be observed, and the overall efficacy and outcome of future clinical trials could be improved [1,2]

Alternative drug testing systems use biomaterials and synthetic scaffolds that are utilized within a perfusion chamber. A dynamic laminar fluid flow system was achieved through the use of a peristaltic pump, tubing, and a medium reservoir. This allows for transport of key nutrients and oxygen throughout the system [3]. However, the scaffolds and biomaterials used to seed the cells, do not contain any vasculature and contain only the trabecular part of the bone. Most of the time only a limited number of cell types are utilized for these studies [4,5,6,7].

In this study, we perfused human femoral heads using a self-designed perfusion bioreactor that sustained cell viability in an ex vivo human femoral head. By cannulating the four main arteries found in the human femoral head, the medial and lateral circumflex arteries and the posterior superior branch, and the posterior inferior branch of the retinacula arteries, we were able to supply the entire femoral head with nutrient-filled media. The media flowing through the bone would provide the bone with the required flow rates, mass transfer, nutrients, pH, and gases. These elements, along with other factors, like temperature, humidity, and removal of toxic waste, will allow for maintaining the femoral head in the dynamic environment required to survive ex vivo.

## 2. Materials and Methods

### 2.1. Bioreactor Design

**Figure 1:**
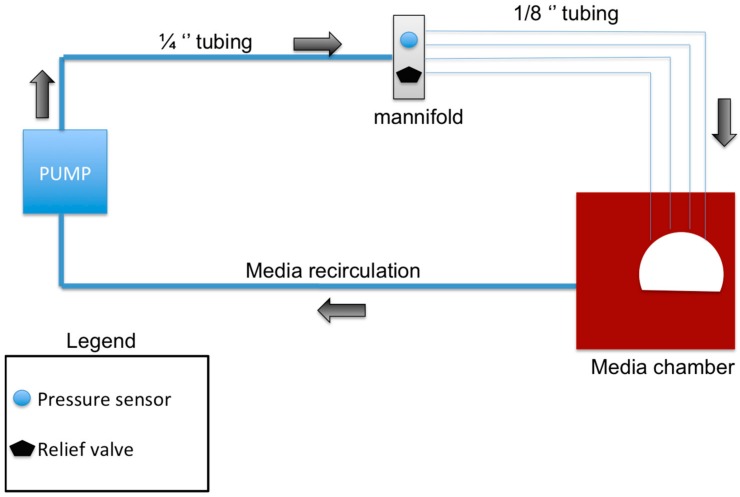
Schematic showing the main components of the bioreactor design: The pump circulates the media in the direction of the black arrows. The manifold splits the single tube into four separate tubes, which correspond to the four main arteries of the femoral head which were cannulated. The media is then recirculated from the media chamber back into the system to form a closed system bioreactor.

**Figure 2:**
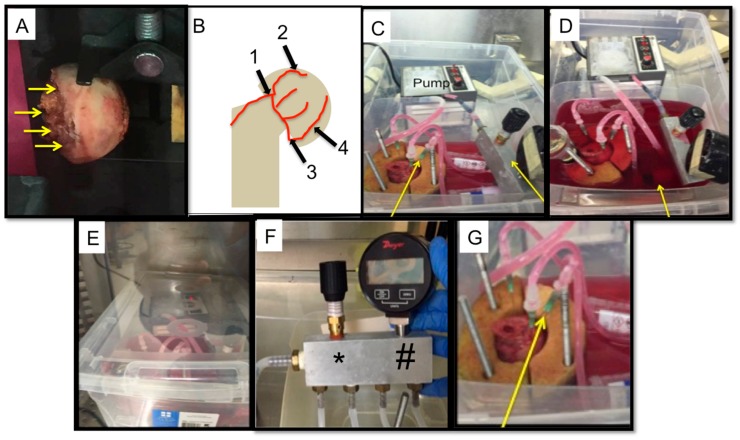
Sample bioreactor design: (**A**) An image of a human femoral head before cannulation and perfusion. The yellow arrows indicate were the perfusion would be attempted. (**B**) A schematic depicting the location of the possible vasculature that was cannulated during perfusion. (1) Lateral cervical ascending artery; (2) posterior superior branch; (3) medial circumflex femoral artery; and (4) posterior inferior branch. (**C**) The bioreactor and all its components, yellow arrows indicate the manifold and the pressure gauge and relief valve. This image was taken prior to completely filling the media chamber with DMEM. (**D**) The bioreactor and all its components. Once the bone is cannulated, the media chamber is filled (designated with a yellow arrow). This image was taken after the chamber was filled up completely. (**E**) The closed system bioreactor. Once the set-up was complete, a lid was placed on the bioreactor system and it was transferred into an incubator, where it remained for the duration of the perfusion experiment. (**F**) Modified manifold design showing the pressure gauge (#) and pressure relief valve (*) which allow continuous monitoring of pressure. (**G**) An enlarged image of the femoral head holder, the arrow demoting one of the four needles cannulating the femoral head.

The perfusion bioreactor was devised to be a closed system. The peristaltic pump P1 (General Electric, Piscataway, NJ, USA) was used to conserve the constant flow of media throughout the bioreactor. A total of 1.75 L of Dulbecco’s Modified Eagle’s Medium (Corning, New York, NY, USA), (with 10% (*v*/*v*) fetal bovine serum (FBS) (Gemini Bio-Products, West Sacramento, CA, USA), 1% (*v*/*v*) penicillin/streptomycin (Gemini Bio-Products, West Sacramento, CA, USA), and 1% antibiotic-antimycotic (Gemini Bio-Products, West Sacramento, CA, USA)), was used to perfuse the system for 12 h. Figure 1 illustrates the flow system of the bioreactor. A peristaltic pump was used to create a perfusion rate of 6–8 mL/min. The pump was connected to the manifold with silicone tubing (ID 1/4" × 3/8” wall thickness) and a final image of the Bioreactor system can be seen in Figure 2A. The manifold branched into four tubes (3/16” ID), which were connected to 21 ½ gauge hypodermic needles that cannulate the femoral head arteries and allow media to perfuse the bone sample (Figure 2C,D). A pressure gauge and relief valve were added to the manifold to maintain a constant pressure and to avoid pressure build-up or backflow (Figure 2B). A pressure range of 0.1–1.1 psi was maintained to mimic the flow pressures in bone. To allow a continuous and sterile exchange of gases, a Breathe Easy® gas permeable sterile film was used (USA Scientific, Ocala, FL, USA). The direction of flow is as follows: from the peristaltic pump, to the manifold, to the femoral head vasculature, from the femoral head vasculature into the media chamber, then back into the pump.

### 2.2. Pre-Soaking Assay to Reduce Pressure Build-Up within the Femoral Heads

Bovine femoral heads were collected from Herman’s Meat Shop in Newark, DE, USA and were incubated in varying concentrations of mannitol (5% mannitol (*w*/*v*) for one hour, 10% mannitol (*w*/*v*) for one hour, 15% mannitol (*w*/*v*) for one hour, and 10% mannitol (*w*/*v*) for two hours) or (0.15% heparin for one hour, 0.0025% of heparin for one hour, and 0.15% of heparin for two hours). All solutions were created in DMEM (Corning, New York, NY, USA), supplemented with 10% (*v*/*v* fetal bovine serum (FBS) (Gemini Bio-Products, West Sacramento, CA, USA), 1% (*v*/*v*) penicillin/streptomycin (Gemini Bio-Products, West Sacramento, CA, USA), and 1% *Antibiotic-Antimycotic* (Gemini Bio-products, West Sacramento, CA, USA).

### 2.3. Villanueva Stain to Show Viable Perfusion

It was important to determine whether DMEM was flowing through the vasculature of the bovine femoral head. Therefore, a 1% (*v*/*v*) solution of Villanueva stain was prepared using 1X PBS, which was run through the bioreactor for a total of 12 h. After 12 h, the samples were sliced into 2–2.5 mm slices using a modified tile saw and the stain was compared to an image of a control bone (that was incubated in the same solution, but not perfused). The Villanueva stain determined the viable perfusion in the femoral head, through the vasculature.

### 2.4. Cell Viability Assay with Mannitol and Heparin

Cell viability was also tested using CellTrace™ Calcein red-orange AM. A Hoechst nuclear stain was also used in order to determine total number of cells, as it stains the nucleus of both live and dead cells. C_2_C_12_ cells were used and treated with varying concentrations of both mannitol (5%, 10%, and 15%) and heparin (0.15% and 0.0025%) for one or two hours. C_2_C_12_ cells in DMEM supplemented with 10% FBS, and 1% penicillin/streptomycin at 37 °C in an environment of 5% CO_2_/95% air were grown. Once ready for treatment, the cells were seeded at 1.0 × 10^5^ cells/mL in a 24 well plate for 12 h before treatment. The growth media was then replaced and supplemented with 1 µM of Calcein red-orange AM in addition to the designated treatment. After the corresponding time point (either one or two hours post treatment) the cells were stained with Hoechst for 2.5 min at room temperature and immediately counted for cell viability. Percent viability was then calculated. This was a standard 2D culture experiment.

### 2.5. Femoral Head Cannulation and Perfusion

Human femoral heads were obtained from female patients with osteoporosis who had undergone total hip replacement surgery at Christiana Care Hospital in Newark, DE, USA. The specimens were kept at 4 °C until use. The specimens were incubated in 10% (*w*/*v*) mannitol in DMEM. Then, a surgical scalpel was used to cut back the neck of the femoral head, which uncovered the four main arteries (or cannulation sites). If cannulation was obstructed, a 21 gauge, 1-1/2-inch needle drill bit was used to hold the needle in its place. To avoid introducing air bubbles into the bone, the tubing was not attached to the cannulation sites until the media was thoroughly pushed through the tubing. The bioreactor was then run for 12 h. After 12 h, the bioreactor was stopped and the samples were processed. The control specimen was an osteoporotic femoral head that was not perfused, but was kept in the same DMEM medium for 12 h in order to determine diffusion of DMEM into the sample.

### 2.6. Parameters Affecting the Uptake of Nutrients and Media Flow

Changes in flow, pressure, glucose levels, and pH of the media were determined because these parameters are important in regards to successful perfusion. If the pressure increased or decreased, this would mean that media was coagulating within the bone. If the glucose levels were to decrease this would signify that the cells within the bone were still alive and taking up vital nutrients. pH was checked to verify that no contamination had occurred, as increases in pH have been linked to contamination [8]. A pressure gauge (Dwyer Instruments, Michigan City, IN, USA) was used and readings were taken every hour in triplicate, pH was measured hourly and was approximated using pH paper. Glucose was measured every three hours using a One Touch Glucose Monitor (LifeScan, Milpitas, CA, USA).

### 2.7. Obtaining Human Femoral Head Bone Slices

Once the bioreactor was stopped and the human femoral head was removed, cores (using a 3/8” drill bit core, cores were 1.5–2 cm in length) of the specimen were taken down the midsagittal plane and immediately placed in DMEM. The femoral head was contained in 1X PBS in order to avoid overheating while the core was taken. These cores were then sectioned using a miter saw (also in 1X PBS). Three slices (about 500 microns) were obtained from the top, middle, and bottom of the core and labeled as such. This was to determine the stain penetration in comparison to perfusion versus diffusion.

### 2.8. Image Analysis of Ex Vixo Cell Viability in Human Femoral Heads

Images of the human bone slices were taken using LSM 880 Zeiss Microscope (Zeiss, Oberkochen, Germany) with an LD LCI plan-apochromat 25×/0.8 l mm korr DIC M27 objective (Zeiss, Oberkochen, Germany). Two lasers (561 nm at 10–22% power and 405 nm at 1.1–1.8% power) were used and images were taken as a Z-stack. A spectral scan was run and collected on a control bone that was stained with 1 µM of Calcein red-orange AM (Calcein acetoxymethyl ester). This was to establish the spectral peak of the dye (590 nm emission spectrum) and was then set as the control peak. Linear mixing was used, in order to identify the defined spectrum and remove autofluorescence of the stain (in some cases fingerprinting technology was also used).

### 2.9. Statistical Analysis

When comparing two groups, a Student’s *t*-test was used with *p* < 0.05 denoting significance. Comparisons of more than two datasets were analyzed using a one-way ANOVA with a Tukey Kramer post-hoc test. Statistical significance was shown if *p* < 0.05.

## 3. Results

### 3.1. Ten Percent Mannitol is the Optimum Pressure Stabilizing and Cell Efficient Supplement for Perfusion Bioreactor Medium

**Figure 3:**
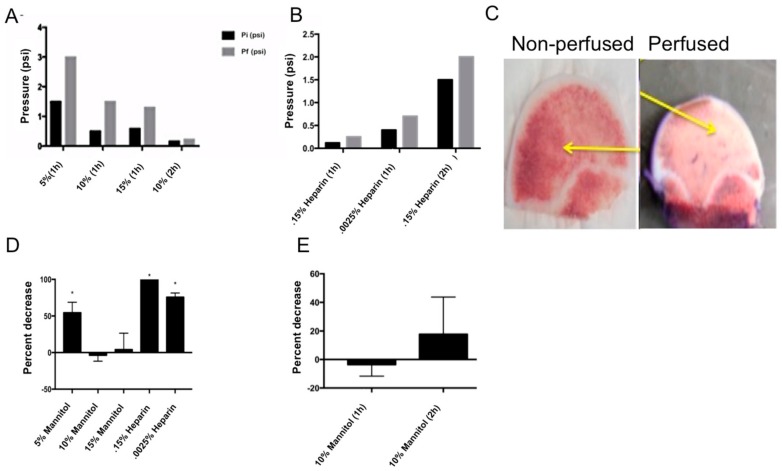
Optimizing perfusion conditions: (**A**) Pre-soaked bovine femoral heads with varying concentrations of mannitol in DMEM. Initial pressure (Pi) and final pressure (Pf) readings were recorded. The two-hour, 10% mannitol soak showed consistent physiological pressure. (**B**) Pre-soaking bovine femoral heads with varying concentrations of heparin in DMEM. Again, initial pressure (Pi) and final pressure (Pf) readings were recorded. The one-hour soak in 1500U (0.15%) and 2500U (0.0025%) of heparin showed consistent physiological pressure readings. (**C**) Testing perfusion versus diffusion using 1% concentration of Villanueva stain in 1X PBS with a heparin presoak. The first image shows a non-perfused bovine femoral head, while the second image is a perfused bovine femoral head. The yellow arrows indicate the intact vasculature through which the Villanueva stain was present in the perfused bone, verse the vasculature that was not stained in the non-perfused bone. (**D**) C_2_C_12_ cell viability test with varying concentrations of mannitol or heparin, one hour after treatment. Both concentrations of mannitol (10% and 15%) showed no significant decrease in the percentage of viable cells (*p* < 0.05). The 0.15% heparin treatment resulted in a significant increase in cell death after one hour. (**E**) C_2_C_12_ cell viability test with 10% mannitol after one and two hours. There was no significant decrease in viable cells after either time point.

Human femoral heads contain several cell types in their native environment, including the vasculature. Therefore, they serve as an ideal test system to study the interaction of potential therapeutics with cells. The drugs can be perfused through the vasculature. However, one drawback is that cells start dying within the bone within 12–24 h after removal from the patients [9]. In order to keep the cells viable, nutrients need to be delivered to them through the vasculature. The blood flow through the vasculature maintains a pressure range of 0.1–1.1 psi [2,10]. In addition long-term storage for 12 h of the femoral heads results in blood coagulation, which causes perfusion pressure to increase. To address this, mannitol and heparin, pressure-relieving agents, were used with bovine femoral heads in order to perfect the parameters of the perfusion bioreactor, before utilizing human femoral heads from patients. Heparin is a known anticoagulant and mannitol has been shown to improve osmotic regulation in perfusion bioreactors [11,12]. To determine the efficacy of mannitol and heparin on pressure, prior to perfusion the femoral head was soaked at different time points (one or two hours) and at different concentrations of either mannitol (5%, 10%, 15%) or heparin (0.15% and 0.0025%). The time points were initially selected due to heparin’s fast-acting anticoagulant ability. Heparin has been shown to act quickly in the clinic time and time again, which is why it is the preferred method in hospitals and ERs today [13]. Initial and final pressure readings were compared for all time points and varying concentrations (Figure 3A,B). Heparin-treated samples showed an increase in pressure within the femoral head, however, it can be noted that the femoral head was noticeably softer and more brittle after the two-hour heparin soak. Mannitol increased cannulation efficiency and decreased the pressure. The two-hour, 10% mannitol soak yielded the lowest change in pressure (0.1 psi) and also maintained the normal, physiological pressure of blood within the femoral head (0.1 to 1.1 psi); therefore, it was selected.

In order to determine the effect of heparin and mannitol on cell viability, C_2_C_12_ cells were stained for CellTrace™ Calcein red-orange AM and Hoechst. This was completed as a 2D culture to ensure that presoaking femoral heads with mannitol or heparin would not be detrimental to cell viability, as cell viability was the endpoint of our perfusion experiment. C_2_C_12_ cells are a mouse skeletal myocyte cell line that are known to differentiate into osteoblasts, adipocytes, and myocytes. The differentiation into myocytes has been shown to take 3–4 weeks under serum-starved conditions. Since we are utilizing a much shorter timeframe (24 h), the C_2_C_12_ cells would not be a myocyte cell line, but a skeletal cell line [14]. These cells were utilized because it has previously been shown that they act similarly in primary bovine bone cell cultures, when these cells were assessed for mineralization activity [3,15]. The cells were then supplemented with the varying concentrations of mannitol and heparin dissolved in media, as discussed above. Cells supplemented with heparin significantly decreased cell viability after one hour of perfusion (Figure 3C). The samples supplemented with 10% or 15% mannitol (*w*/*v*) had no effect on cell viability after one hour of perfusion. There was no effect on cell viability with 10% mannitol even after two hours of exposure (*p* < 0.05; Figure 3D). Heparin was not used during perfusion (or as the pressure relieving agents) because of the decreased cell viability and the softness/brittleness of the bone. Mannitol was used as the pressure-relieving agent, but only as a presoak. It was not used during the perfusion because mannitol has been shown to increase hypertension [16].

### 3.2. Pressure Remains Constant while Sugar Levels Change in the Perfusion Bioreactor of Human Femoral Heads

**Figure 4:**
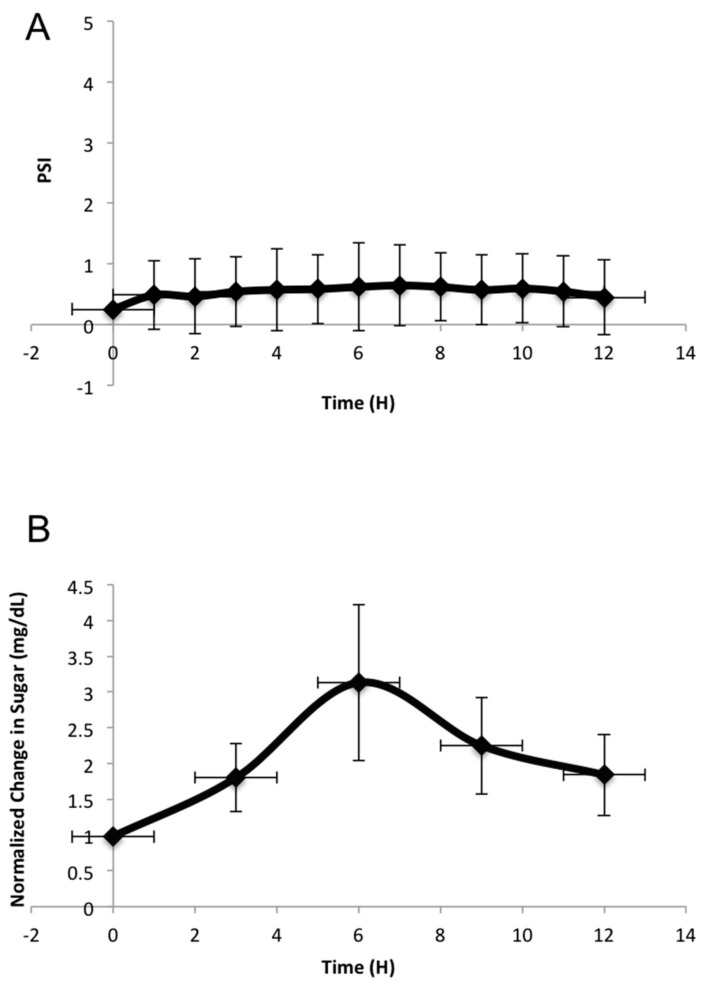
Pressure and sugar changes during 12-h perfusion bioreactor experiments: (**A**) Change in pressure perfused through the femoral head over 12-h bioreactor runs (n = 3). The pressure remained between physiological levels of 0.1 and 1.1 psi and was measured through a pressure sensor. (**B**) Sugar readings were taken every three hours for a 12 h period, while the bioreactor was running and femoral heads were being perfused. Sugar readings from the perfusion experiments were subtracted from an averaged glucose reading of the media, without a femoral head. From there, the sugar readings were normalized to the 0-h time point. This is an average of three independent experiments. All error bars represent standard error.

During the bioreactor experiment, pressure remained relatively constant throughout the 12 h of perfusion, ranging between 0.1 and 1.1 psi (Figure 4A). Sugar levels increased slightly, and then decreased slightly as the 12 h progressed (Figure 4B). This indicates that the mannitol is perfused out of the femoral head into the media and then sugar concentrations decrease due to the consumption of the sugar by the cells within the femoral head. It did take six hours for the sugar concentration to reach its maximum level (3 mg/dL) within the system. Which could have been due to the way in which the glucose was measured. Three 1-mL aliquots of media were removed from the media chamber every three hours to take the sugar readings. The readings were averaged for each time point, for each individual bioreactor run. The media chamber held 1.75 L of media, this large sum of media could explain why there was a gradual increase in sugar, which occurred slowly over the course of the experiment.

### 3.3. Cell Viability is Increased in Perfused Human Femoral Heads

Our perfusion bioreactor uses the vasculature to perfuse media through the femoral head. To compare the effects of vascular perfusion to simple diffusion across the tissue, we used CellTrace^TM^ Calcein red-orange AM (789.55 g/mol) to determine the difference between diffusion and perfusion.

**Figure 5:**
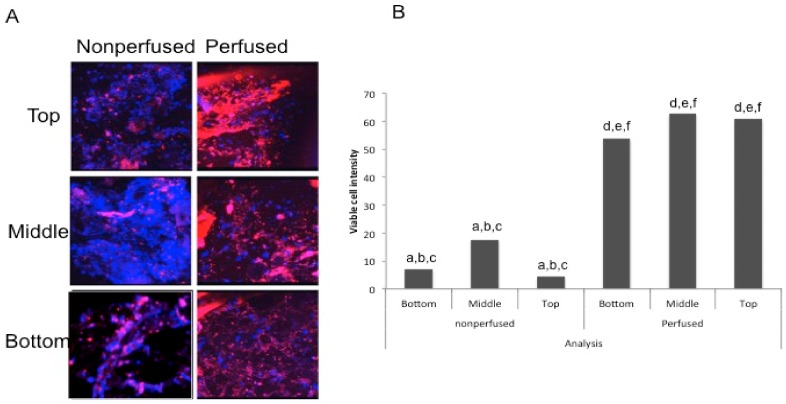
Cell viability after a 12-h perfusion: (**A**) Three-dimensional view of the top, middle, and bottom slices from an internal core of both the perfused and non-perfused femoral heads. A blue Hoechst stain was used to visualize the nuclei of both live and dead cells, while a red Calcein AM stain was used to visualize live cells. (**B**) A graph depicting the intensities of viable cells in both the perfused and non-perfused bone. A z-stack was obtained and analyzed in ImageJ, where the intensity of Calcein was measured, subtracted from the background intensity, and subsequently divided by the number of blue nuclei. All sections of the perfused femoral head had significantly more viable cells than the non-perfused femoral head. This is an average of three independent experiments, using a total of six different femoral heads.

Human femoral heads underwent a 12-h perfusion and were successively cored, sliced, imaged, and analyzed. A control femoral head was also used without any cannulation. It was also sliced and cored in the same manner. Both sets of slices (perfused and non-perfused) were imaged on the Zeiss LSM 880 for live cell imaging, as described in the Methods section. Slices from the top, middle, and lower part of the femoral head were compared for perfusion. Only perfused regions that include live cells will stain. Figure 5A shows live cell imaging of both the perfused and non-perfused femoral head sliced cores. The red stain seen is the Calcein red-orange, indicating live, viable cells. The blue stain seen is a nuclear Hoechst stain, which stains the nucleus of both live and dead cells. The images seen in Figure 5A are a 3D rendering of the bone slices. The slices were taken from the top, middle, and bottom portions of the femoral head. In Figure 5B, the intensity of the stains were quantified in ImageJ and normalized in order to determine if a greater amount of Calcein red-orange stain was observed in the images. This figure indicates that the staining in the perfused slices were significantly different from the non-perfused slices at all levels of the core. These data indicate that vascular perfusion of human femoral heads is much greater than diffusion and the viability of cells was maintained ex vivo. Additionally, it shows that the media is perfused throughout the whole femoral head.

## 4. Discussion

Bioreactors for tissue engineering are still in the early stages of development. A tissue engineering bioreactor must: (1) have even cell distribution throughout the scaffold; (2) satisfy physiologically-relevant factors (like nutrients, oxygen, pH, removal of waste, temperature, and humidity) that is needed by the tissue [17]; (3) aid in the mass transport of medium throughout the scaffold; and (4) integrate required physical parameters (like the flow rate of the blood within bone, pressure, fluid shear stress, resistance, and compliance). The bioreactor should also be effortlessly monitored, controlled, and sterile, in addition to the results being reproducible [1]. Our perfusion bioreactor was designed to address these parameters. Silicone tubing was used throughout the system because it is a commonly used biomaterial in implants, biocompatible, and can be autoclaved at 121 °C for 15 min [18]. A manifold was designed using stainless steel because previous studies have shown that long-term use does not increase toxicity [17]. Within the manifold there were a pressure gauge and pressure relief valve. These were used to adjust the pressure levels if it was outside the normal, physiological range of 0.1–1.1 psi [19,20]. Syringe needles (21 ½ gauge) were used to cannulate the bone, because their width (0.5 mm) matched the inner diameter of four main arteries (0.5–8.5 mm).

The perfusion flow rate within the femoral heads were maintained at 6–8 mL/min. This is comparable to the in vivo blood flow rate in the bone, which is approximately 5–20 mL/min [21]. We ensured bone viability through perfusion of media into the pre-existing vasculature found within the femoral head. Calcein red-orange AM is a red orange dye that is intrinsically fluorescent and can permeate through the plasma membrane of live cells, in part, due to its a small molecular weight (789.55 g/mol). A concentration of 100 nM was mixed into the media that was perfused through the bone.

In order to extend the lifespan of the femoral head ex vivo, several trouble-shooting experiments were conducted in order to optimize the efficacy of our bioreactor. Pressure-relieving agents (mannitol and heparin) were tested in order to ensure continuous media flow through the femoral head and the results are shown in Figure 3A,B. Heparin inhibits the activity of thrombin, which is accountable for removing blood clots. However, heparin also activates the enzyme lipoprotein lipase in the blood. Heparin-soaked bones were noticeably softer than the mannitol-soaked bones. The previously described enzymatic activity is a possible explanation for the softening of the bone [11]. Mannitol increases the osmolarity of the medium and effectively removed clots formed, aiding in keeping the flow of media constant, without altering the integrity of the bone [22]. Continued, long-term use of mannitol has been shown to increase hypertension, therefore, shorter time points were selected [16]. The effect of mannitol and heparin on cell viability demonstrated that mannitol was the more effective supplement (Figure 3C,D). In addition, a Villanueva stain was used in order to show the successful and viable perfusion through a bovine femoral head when compared to a non-perfused bovine femoral head. This stain showed that the perfusion had occurred successfully through the vasculature, and that apoptosis due to re-perfusion injury was minimal.

The human femoral heads used were obtained from female osteoporotic patients between the ages of 80–90 and were collected from Christiana Care Hospital within 24 h of hip arthroplasty surgery. Previous in vitro experiments using human specimens have shown the presence of some live cells within 24 h of procurement [23], therefore, it would be possible to obtain live cell images from a perfused femoral head after 12 h of perfusion. The presence of live cells and the perfusion of bone are shown in Figure 5A. The Calcein red-orange stain was more apparent throughout the perfused bone, which was not observed in the control. The pressure was maintained between 0.1–1.1 psi, while the sugar levels increased, and then subsequently decreased, over time. The increase in sugar could be attributed to the mannitol being released from the femoral head, and the subsequent decrease could suggest that it was being utilized by the cells of the femoral head (Figure 4A,B). The glucose meter utilized could detect multiple types of sugar (including mannitol), which could explain why there was an increase.

Limitations of this study include the number of trials conducted (three), the tissue (femoral head) used, and the time period the femoral head was perfused. In the future more trials across different bone tissue types should be investigated in order to obtain reliable data regarding the use of a perfusion bioreactor to study bone in vivo. The method in measuring sugar concentrations could also be improved upon, since a commercial glucose monitor was used, and could be attributed to the unexplained sugar spike at six hours. In addition, the bioreactor should be run for a longer period of time (24 h or 48 h) to confirm its effectiveness in maintaining cell viability ex vivo. Femoral heads from female patients aged 80–90 years old were used and, in the future, more trials should include femoral heads from both male and female patients, as well as patients at varying ages, in order to eliminate possible confounding variables.

In conclusion, this study shows that human femoral heads can be used as a biological platform and bone cell viability can be maintained ex vivo. The successful perfusion in human femoral heads was shown through the viability of cells 12 h post perfusion (Figure 5A,B). This is a potential drug-testing model that could be invaluable in the future for osteoporotic therapeutic testing.
